# Research Trends in General Medicine: A Domain-Based Bibliometric Analysis of Three Leading Generalist Journals

**DOI:** 10.7759/cureus.98293

**Published:** 2025-12-02

**Authors:** Keigo Takeuchi, Kota Sakaguchi, Rintaro Kaminishi, Takashi Watari

**Affiliations:** 1 Family Medicine, Shimane University, Izumo, JPN; 2 General Medicine, Shimane Prefectural Central Hospital, Izumo, JPN; 3 Integrated Clinical Education Center, Kyoto University, Kyoto, JPN; 4 General Medicine Center, Shimane University Hospital, Izumo, JPN

**Keywords:** bibliometrics, family medicine, general internal medicine, hospital medicine, research trends

## Abstract

To advance the field of general medicine, it is essential to characterize its research landscape. General medicine is divided by clinical settings into domains such as general internal medicine, hospital medicine, and family medicine, but their research characteristics remain largely unexplored. This study aims to analyze articles published in three leading general medicine journals - Journal of General Internal Medicine (JGIM), Journal of Hospital Medicine (JHM), and Annals of Family Medicine (AFM) - to identify the primary research focus of each domain and describe article themes. We conducted a cross-sectional bibliometric analysis of original articles published between January 1 and December 31, 2023. Articles were classified into seven research themes: "Clinical Research," "Public Health/Preventive Medicine/Epidemiology," "Basic Research," "Medical Education," "Quality and Safety of Healthcare," "Health Services Research," and "Others." A total of 717 original articles were included: 591 from JGIM, 68 from JHM, and 58 from AFM. The most frequent themes were "Public Health, Preventive Medicine, and Epidemiology" in JGIM (43.1%) and "Quality and Safety of Healthcare" in JHM (64.7%). In AFM, research topics were distributed among "Public Health, Preventive Medicine, and Epidemiology" (29.3%), "Quality and Safety of Healthcare" (24.1%), and "Health Services Research" (20.7%). This study revealed that general medicine, while diverse, has research focuses that differ by journal. These findings provide an evidence base for future strategic research planning, topic selection, and the development of domain-specific research support systems.

## Introduction and background

Research is essential for the maturation of medicine as an academic discipline, acting synergistically with clinical practice, a specialized body of knowledge, and education [1]. Within this synergy, research plays a pivotal role in strengthening the academic foundation of general medicine [2]. For the purpose of this study, we define general medicine as comprising three major domains based on clinical setting and academic affiliation: general internal medicine (GIM), hospital medicine (HM), and family medicine (FM). However, compared with other specialties, research in these generalist fields faces significant limitations. Specifically, academic GIM is challenged by resource constraints and defining its unique research scope [3,4], HM research often encounters barriers such as a lack of protected time and mentorship [5], and FM research faces substantial global challenges in infrastructure and funding [6]. Despite the urgent need to expand the evidence base for common conditions managed by generalists, the current research base remains insufficient.

International organizations such as the World Organization of Family Doctors (WONCA) and the World Health Organization (WHO) have repeatedly emphasized the importance of research in primary care, recognizing it as an essential component for achieving high-quality, sustainable healthcare systems [4]. Consequently, the volume of academic publications related to general medicine has been increasing in many countries; however, global differences in academic publication volume and research infrastructure persist between regions [5,6].

This situation is partly due to a historical tendency within general medicine to apply research findings from other specialties to its clinical and educational practices, with a limited amount of original research focusing on general medicine. Furthermore, structural constraints, including insufficient time, funding, and mentorship for research, coupled with a lack of comprehensive strategies to promote research, have contributed to the limited amount of research in the field. Although the research questions within primary care are diverse, they lack a well-organized overall landscape, making it difficult to establish clear research priorities [7].

Although previous reports have described research trends within general medicine as a whole or in specific domains, even at an international level, very few studies have systematically compared and analyzed research themes by both domain and journal. Consequently, the key research areas in general medicine, as well as the commonalities and differences among its domains, are poorly understood [8]. Therefore, this study focused on the three primary domains of general medicine (GIM, HM, and FM) and aimed to identify the primary research focus of each domain and the commonalities and differences in their thematic trends by analyzing original articles published in three leading international general medicine journals.

## Review

Methods

Study Design and Search Strategy

This study was conducted as a cross-sectional bibliometric analysis of research articles published in three leading general medicine journals: the Journal of General Internal Medicine (JGIM), the Journal of Hospital Medicine (JHM), and the Annals of Family Medicine (AFM). The methodology adhered to the generally accepted standards for systematic literature reviews, particularly concerning article identification and selection. The analysis targeted articles from leading international academic journals representing the three main domains of general medicine [7]. Journals were selected based on three criteria: (1) international recognition, (2) specialization in the target domain, and (3) having a Journal Impact Factor (JIF) [8]. The search was performed on the PubMed database for all articles published between January 1 and December 31, 2023, using the specific journal names and publication dates as search limits.

Inclusion and Exclusion Criteria

The inclusion criteria for article selection were as follows: (1) publication in one of the three selected journals; (2) publication date between January 1 and December 31, 2023, as indexed in PubMed; and (3) article type being original research, concise research report, or review (e.g., systematic reviews, narrative reviews, and scoping reviews). Opinion pieces and commentaries such as editorials, perspectives, viewpoints, and brief reports were excluded. Articles where the full-text was not accessible or retrievable (or required purchase) and where final classification was impossible by consensus were excluded from the analysis to ensure the replicability and reliability of the data analysis.

Data Extraction and Classification

Two authors (K.T. and R.K.) independently reviewed the titles and abstracts of the extracted articles and classified them based on the research theme. Following the methodology of Watari et al., we used the following seven categories: (1) Clinical Research, (2) Public Health/Preventive Medicine/Epidemiology, (3) Basic Research, (4) Medical Education, (5) Quality and Safety of Healthcare, (6) Health Services Research, and (7) Other [9]. For articles whose content overlapped multiple research theme categories, the final classification was determined by the primary focus of the article as assessed by the consensus of the reviewers, thereby ensuring that each article was assigned to only one distinct theme category. This classification process aimed to minimize bias and enhance the clarity of the bibliometric data.

Ethical Considerations

This study was a bibliometric analysis based solely on publicly available published articles. The research “subjects” were journal articles. As no humans or animals participated in the research, the study was exempt from institutional review board review and approval.

Results

Article Selection

**Figure 1:**
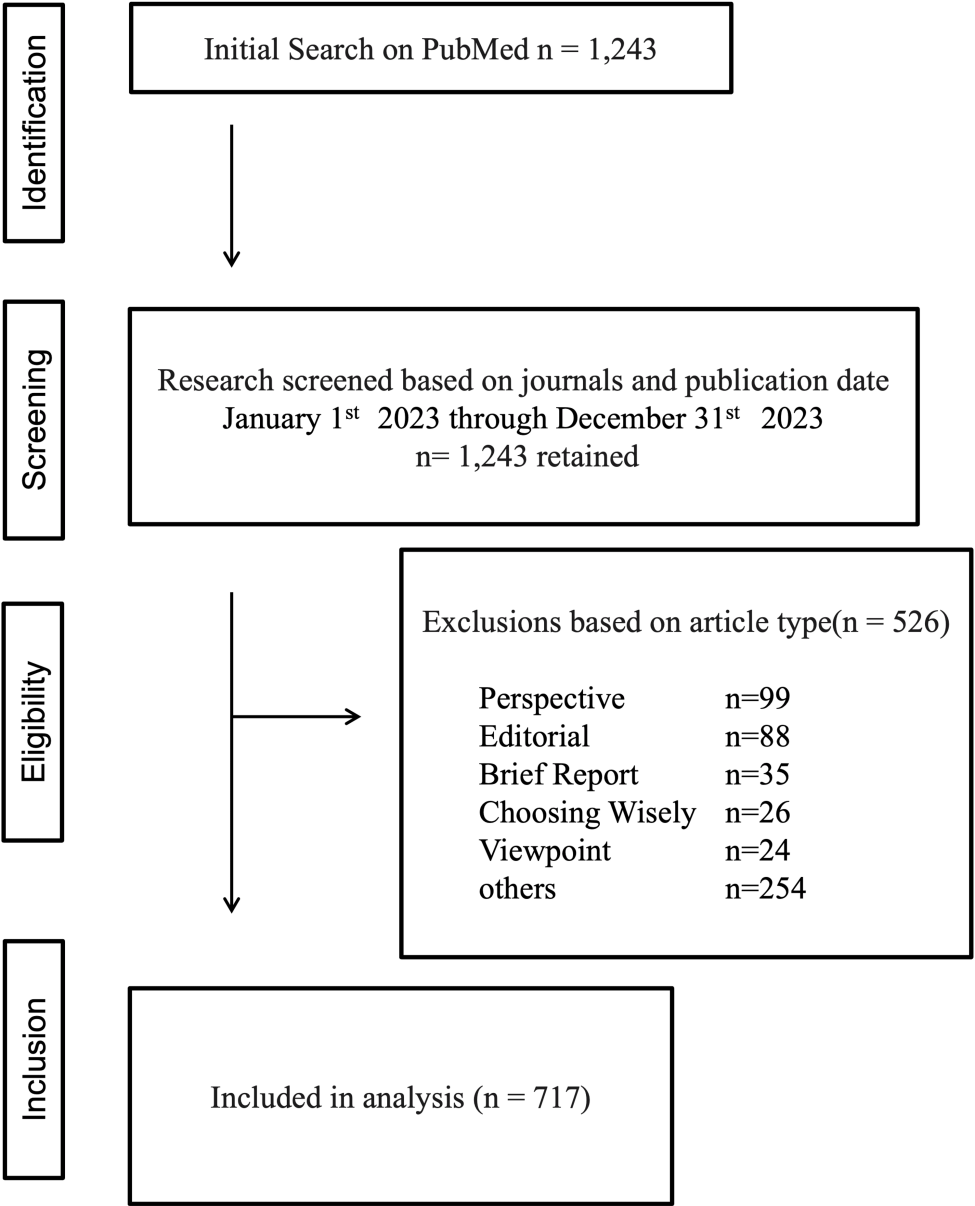
Total number of articles and selection process. Articles were identified from three target journals indexed in PubMed and screened by article type. Editorials, perspectives, viewpoints, and other non-research articles were excluded. Eligible publications included original research articles, concise research reports, and reviews.

During the study period, a total of 1,243 articles from the three target journals were identified in PubMed. By journal, 823 articles (66.2%) were from the JGIM, 276 (22.2%) were from the JHM, and 144 (11.6%) were from AFM. Of these, 527 non-research articles, such as editorials and perspectives, were excluded, resulting in a final sample of 717 articles for analysis (Figure 1), of which 591 (82.4%) were from JGIM, 68 (9.5%) were from JHM, and 58 (8.1%) were from AFM.

Types and Distribution of Articles

Overall, the majority of publications (79.1%) were original research, with concise research reports and reviews accounting for 14.6% and 6.3% of publications, respectively (Table 1). Of the 591 articles published in JGIM, 453 (76.6%) were original research articles, 105 (17.8%) were concise research reports, and 33 (5.6%) were reviews. Of the 68 articles published in JHM, 62 (91.2%) were original research articles, and six (8.8%) were reviews, with no concise research reports. Of the 58 articles published in AFM, 52 (89.7%) were original research articles, and six (10.3%) were reviews, with no concise research reports.

**Table 1:** Article types by journal. Data are presented as n (%) otherwise indicated. JGIM: Journal of General Internal Medicine; JHM: Journal of Hospital Medicine; AFM: Annals of Family Medicine

Article type	Journals			
	JGIM, n (%)	JHM, n (%)	AFM, n (%)	All, n (%)
Original research	453 (76.6)	62 (91.2)	52 (89.7)	597 (79.1)
Concise research report	105 (17.8)	0 (0)	0 (0)	105 (14.6)
Review	33 (5.6)	6 (8.8)	6 (10.3)	45 (6.3)
Total	N = 591	N = 68	N = 58	N = 717

Distribution of Research Themes

The classification of research themes by journal is presented in Table 2. Clear differences in thematic focus were observed among the journals. In JGIM, “Public Health/Preventive Medicine/Epidemiology” was the most frequent theme, accounting for 255 articles (43.1%). This was followed by “Quality and Safety of Healthcare” (110 articles, 18.6%) and “Clinical Research” (98 articles, 16.6%). “Health Services Research” (66 articles, 11.2%) and “Medical Education” (60 articles, 10.2%) were also well-represented. Only two articles (0.3%) were classified as “Basic Research,” and none were categorized as “Other.” In JHM, “Quality and Safety of Healthcare” was the predominant theme, with 44 articles (64.7%), followed by “Public Health/Preventive Medicine/Epidemiology” (12 articles, 17.6%). Less than 10 articles each were published on “Medical Education” (six articles, 8.8%), “Clinical Research” (three articles, 4.4%), “Health Services Research” (two articles, 2.9%), and “Basic Research” (one article, 1.5%). In AFM, research themes were more evenly distributed. The main categories were “Public Health/Preventive Medicine/Epidemiology” (17 articles, 29.3%), “Quality and Safety of Healthcare” (14 articles, 24.1%), “Health Services Research” (12 articles, 20.7%), and “Clinical Research” (10 articles, 17.2%). Only five articles (8.6%) were published on “Medical Education,” and no articles were classified as “Basic Research” or “Other.”

**Table 2:** Research themes by journal Data are presented as n (%) otherwise indicated. JGIM: Journal of General Internal Medicine; JHM: Journal of Hospital Medicine; AFM: Annals of Family Medicine

Research theme	Journals			
	JGIM, n (%)	JHM, n (%)	AFM, n (%)	All, n (%)
(1) Clinical Research	98 (16.6)	3 (4.4)	10 (17.2)	111 (15.5)
(2) Public Health/Preventive Medicine/Epidemiology	255 (43.1)	12 (17.6)	17 (29.3)	284 (39.6)
(3) Basic Research	2 (0.3)	1 (1.5)	0 (0)	3 (0.4)
(4) Medical Education	60 (10.2)	6 (8.8)	5 (8.6)	71 (9.9)
(5) Quality and Safety in Healthcare	110 (18.6)	44 (64.7)	14 (24.1)	168 (23.4)
(6) Health Services Research	66 (11.2)	2 (2.9)	12 (20.7)	80 (11.2)
(7) Other	0 (0)	0 (0)	0 (0)	0 (0)
Total	N = 591	N = 58	N = 68	N = 717

Discussion

This study classified the research themes of 717 articles from three leading, relatively high-impact, general medicine journals into three major domains of general medicine: GIM, HM, and FM. The results revealed distinct research priorities in each domain, highlighting the different interests and practices specific to each field. Specifically, JGIM (GIM) focused on “Public Health/Preventive Medicine/Epidemiology,” JHM (HM) prioritized “Quality and Safety of Healthcare,” and AFM (FM) showed a relatively even distribution across multiple domains.

Research Characteristics of Each Domain

In this section, we discuss the research characteristics of each journal in relation to its background and clinical context.

JGIM (GIM): In JGIM, “Public Health/Preventive Medicine/Epidemiology” was the most frequent theme, with approximately 2.3 times more articles than those on “Quality and Safety of Healthcare,” the second most frequent theme. According to a WHO report, approximately 80% of heart disease, stroke, and type 2 diabetes can be prevented through lifestyle modifications, and general internists are expected to engage in preventive interventions at the population level [10,11]. Our findings appear to reflect this role. Studies on “Quality and Safety of Healthcare” were also relatively common. GIM operates in a high-volume, high-turnover environment, where risks such as medication errors and diagnostic delays are a concern [12]. Initiatives to improve safety are thus a clinical priority and a corresponding research subject [13]. The relatively low number of “Clinical Research” articles may be attributed to the challenges of conducting research in busy clinical settings, despite the recognized need [14], due to constraints on human resources, time, and resources [15]. Research on “Health Services” and “Medical Education” was also limited, possibly due to the technical and ethical challenges of implementing telehealth and AI technologies [16-18] and the difficulty of applying medical education research findings to clinical practice [19].

JHM (HM): In JHM, “Quality and Safety of Healthcare” was the most common theme, with approximately 3.7 times more articles than those on “Public Health/Preventive Medicine/Epidemiology,” the second-most frequent theme. Given that approximately one-third of hospitalized patients experience preventable adverse events [20], HM physicians are routinely required to manage safety and improve the quality of care. This research trend aligns with the core competencies for hospitalists outlined by the U.S. Society of Hospital Medicine in 2017 [21]. These competencies emphasize medical safety, quality improvement, appropriate use of medical resources, management, team approaches, interprofessional collaboration, patient education, and care transitions. This suggests that hospitalists are expected to function as “system-managing physicians” responsible for optimizing the entire ward. Indeed, hospitalists dedicate significant time to coordinating activities like teamwork and rounds [22], and these clinical, educational, and operational practices are reflected in the “Quality and Safety of Healthcare” research category. In contrast, the proportion of “Public Health/Preventive Medicine/Epidemiology” research was relatively low. This may be related to the difficulty of intervening on social determinants of health in a hospital setting and the challenges of evaluating post-discharge interventions in a research design [23]. However, the existing preventive medicine articles in JHM primarily focus on acute and system-based issues, such as infection control, delirium prophylaxis, and optimization of post-discharge medication reconciliation, which clearly differentiate them from the broad, population-level health promotion topics emphasized in JGIM. Thus, the research trends in JHM reflect not only the operational characteristics of the clinical setting but also the core competencies required of hospitalists, offering important insights for education and workforce development.

AFM (FM): In AFM, research themes were relatively evenly distributed, with “Public Health/Preventive Medicine/Epidemiology,” “Quality and Safety of Healthcare,” “Health Services Research,” and “Clinical Research” being the primary areas of interest. This multifaceted research focus is likely associated with the diverse roles required of FM physicians. WONCA lists core competencies for family physicians as including (1) psychosocial assessment, (2) health education and behavior change support, (3) stress management and behavioral activation, (4) remote interventions using the internet, (5) problem-solving approaches, and (6) comprehensive care including interprofessional collaboration [17]. These competencies align with our theme categories (e.g., public health, health services, quality, and safety), indicating that the practice of FM readily translates into research themes. For example, in public health and preventive medicine, family physicians routinely provide health education and prevent lifestyle-related diseases in their communities [24]. In health services research, they are engaged in implementing telehealth to improve continuity and accessibility of care, conducting home visits, and building comprehensive care systems [25,26]. From a quality and safety perspective, achieving patient-centered care, supporting patient empowerment, and improving care systems through interprofessional collaboration are also potential research topics [27,28]. Conversely, the number of medical education and basic research articles in AFM was relatively small, which may be due to resource constraints and regional disparities in educational opportunities within the field of FM [29,30]. In summary, the diverse research fields in AFM reflect the comprehensive and collaborative role of family physicians and are a logical outcome consistent with international educational goals and practical competencies in FM.

Strategic Implications for Domain Development

These distinct trends provide a clear foundation for strategic planning within general medicine. For instance, research strategies should now focus on addressing the identified gaps and strengthening existing strengths: enhancing patient safety research in HM, diversifying clinical trials in GIM, and bolstering health services research in FM. This targeted approach is crucial for accelerating evidence generation across the three distinct clinical domains.

Strengths and Limitations

This study has three main strengths. First, to our knowledge, it is the first to quantitatively summarize and compare research themes across the three major domains of general medicine (GIM, HM, and FM). Although these domains are often separated academically and educationally, our study helps to provide a holistic view of the academic field of general medicine by elucidating their commonalities and differences. Second, by clarifying the research focus of each journal, this study offers practical and structural insights for future research strategies, mentoring of junior researchers, and allocation of research resources. Third, it provides generalist physicians who have not yet engaged in research with an overview of representative research themes and underexplored areas within their respective domains.

This study also has several limitations. First, our analysis was limited to three journals. Including other literature databases or related international journals (e.g., BMC Family Practice, Medical Teacher) might reveal different research trends. Second, the analysis was restricted to a single year, 2023, and the findings may be influenced by the unique societal context of the COVID-19 pandemic. The cross-sectional design does not allow for the examination of trends over time, and this limitation should be interpreted with caution. Third, the research theme classification we employed was pragmatic and exclusive, requiring each article to be assigned to a single category. This approach has inherent limitations in fully capturing the multi-layered and cross-disciplinary nature of some research. Future research should consider expanding the analysis period to conduct longitudinal studies tracking thematic changes over time and undertaking more comprehensive analyses that include a wider range of journals and databases. Furthermore, developing algorithmic theme classification and integrating hypothesis-driven research to strengthen quantitative evidence would be valuable next steps for advancing research in the field of general medicine.

This study classified the research themes of 717 articles from three leading, relatively high-impact, general medicine journals into three major domains of general medicine: GIM, HM, and FM. The results revealed distinct research priorities in each domain, highlighting the different interests and practices specific to each field. Specifically, JGIM (GIM) focused on “Public Health/Preventive Medicine/Epidemiology,” JHM (HM) prioritized “Quality and Safety of Healthcare,” and AFM (FM) showed a relatively even distribution across multiple domains.

## Conclusions

This bibliometric analysis of articles published in 2023 in three leading international journals focusing on GIM, HM, and FM revealed distinct research priorities for each domain. These findings demonstrate that general medicine is a diverse academic field with domain-specific interests and challenges. Specifically, GIM focuses heavily on “Public Health/Preventive Medicine/Epidemiology,” HM on “Quality and Safety of Healthcare,” while FM demonstrates a balanced distribution across multiple themes. These findings provide a direct and objective basis for future development. Specifically, domain-specific research strategies should be tailored: GIM should continue to emphasize population health, HM must sustain and refine its patient safety focus, and FM must actively grow its health services and implementation science research. This specificity facilitates targeted topic selection by individual researchers, directing GIM residents toward longitudinal cohort studies, HM residents toward Quality Improvement Projects related to systems of care, and FM trainees toward community-based participatory research. Furthermore, these data inform the creation of domain-specific research support systems, requiring GIM centers to prioritize large-scale data access, HM groups to formalize mentorship for Quality Improvement Projects, and FM departments to allocate dedicated funding for primary care infrastructure studies. This domain-focused approach is essential for establishing mature academic disciplines within general medicine.
